# Airway Management with Cervical Spine Immobilisation: A Comparison between the Macintosh Laryngoscope, Truview Evo2, and Totaltrack VLM Used by Novices—A Manikin Study

**DOI:** 10.1155/2016/1297527

**Published:** 2016-02-29

**Authors:** Dawid Aleksandrowicz, Tomasz Gaszyński

**Affiliations:** ^1^London North West Healthcare NHS Trust, Anaesthetics Department, Northwick Park Hospital, London HA1 3UJ, UK; ^2^Department of Emergency and Disaster Medicine, Medical University of Łódź, 92-209 Łódź, Poland

## Abstract

Airway management in patients with suspected cervical spine injury plays an important role in the pathway of care of trauma patients. The aim of this study was to evaluate three different airway devices during intubation of a patient with reduced cervical spine mobility. Forty students of the third year of emergency medicine studies participated in the study (F = 26, M = 14). The time required to obtain a view of the entry to the larynx and successful ventilation time were recorded. Cormack-Lehane laryngoscopic view and damage to the incisors were also assessed. All three airway devices were used by each student (a novice) and they were randomly chosen. The mean time required to obtain the entry-to-the-larynx view was the shortest for the Macintosh laryngoscope 13.4 s (±2.14). Truview Evo2 had the shortest successful ventilation time 35.7 s (±9.27). The best view of the entry to the larynx was obtained by the Totaltrack VLM device. The Truview Evo2 and Totaltrack VLM may be an alternative to the classic Macintosh laryngoscope for intubation of trauma patients with suspected injury to the cervical spine. The use of new devices enables achieving better laryngoscopic view as well as minimising incisor damage during intubation.

## 1. Background

Airway management and adequate ventilation with simultaneous cervical spine immobilisation form the two most important priorities during the management of trauma patients. The stabilisation of the cervical spine during intubation or, more precisely, during airway management was introduced into the clinical practice in the 1970s. Such an approach significantly reduces secondary neurological damage associated with trauma to the cervical vertebrae [1]. Data available from the studies on healthy anaesthetised volunteers showed reduced movement of the cervical spine by 50% when manual in-line stabilisation (MILS) was applied during intubation [2]. Such an approach reduces the risk of spinal cord injury caused by unstable cervical segments which can result in serious neurological complications including death. Application of MILS during airway management may markedly worsen the laryngoscopic view resulting in difficult intubation. Adverse effects of cervical spine immobilisation include a significantly increased risk of difficult laryngoscopy, an increased time required to intubate a patient, and an increased likelihood of failed intubation [3]. There are available guidelines on airway management in trauma patients in out-of-hospital settings which recommend application of MILS during intubation of the injured [4].

The current approach to airway management in trauma patients with suspected injury to the cervical spine should incorporate the use of alterative (to the classic laryngoscope) airway devices, for example, videolaryngoscope, supraglottic airway devices (SADs), or optical stylets [5–13]. Some of the strategies or techniques, that is, flexible fiberoptic laryngoscopy and nasotracheal intubation, have limited application in trauma victims and in prehospital settings in particular.

The literature on cervical spine immobilisation during simultaneous airway management has up until now been focused on different techniques and devices used by medical professionals of varying levels of experience, for example, trainees in anaesthetics or consultants. The results of such studies may not be objective (risk of selection bias). To our knowledge there are currently no publications that have compared airway (intubation) devices and simultaneous application of MILS by people with no previous experience in advanced airway management, that is, novices.

**Figure 1:**
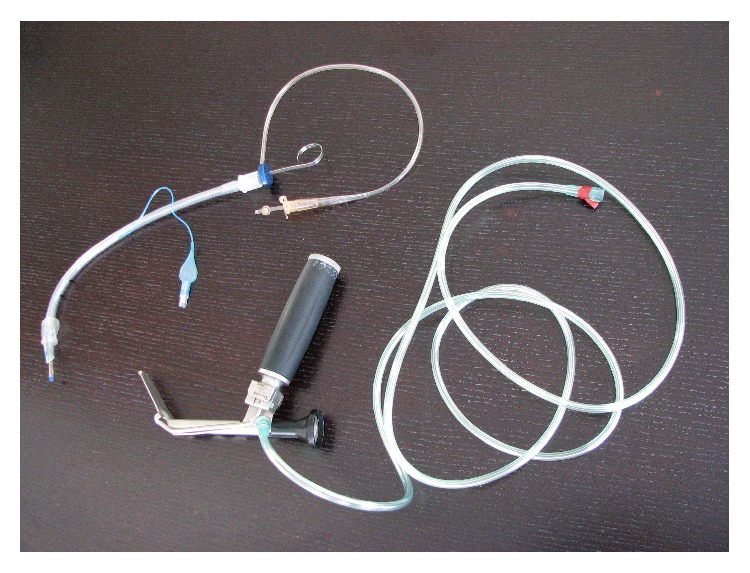


**Figure 2:**
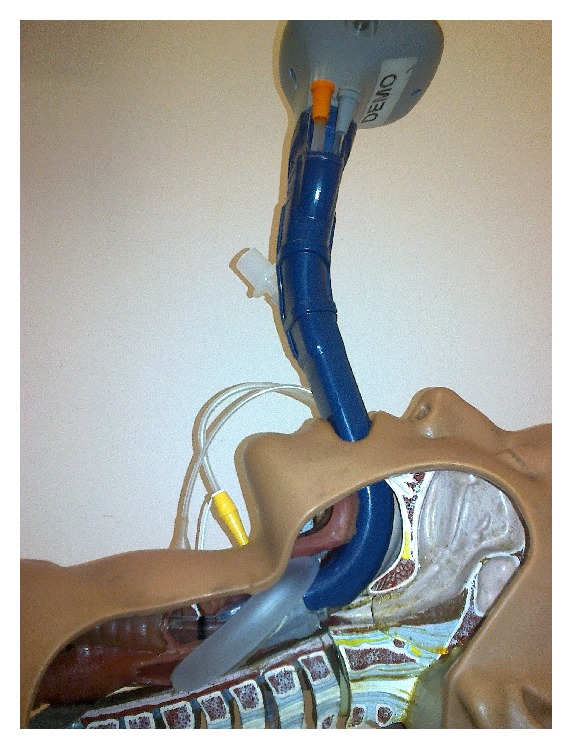


The aim of our study was to evaluate three different airway devices: the classic Macintosh laryngoscope (New Waseem Trading, Sialkot, Pakistan), Truview Evo2 optical laryngoscope (Truphatek International, Netanya, Israel) (Figure 1), and the Totaltrack VLM (Video Laryngeal Mask) device (Figure 2) (Medcom Flow, Viladecans, Barcelona, Spain) in the hands of students without previous intubation experience. The three devices were compared during simulated conditions of restricted/reduced cervical spine movement.

## 2. Materials and Methods

Forty students in their third year of emergency medicine studies at the Medical University of Łódź participated in the study. The majority of participants were female (*n* = 26) compared to male (*n* = 14). All students participating in the study had never used the airway devices under study, did not have previous intubation skills, and were novices in this field. A 15-minute lecture was delivered before the start of the study. It explained how to use each of the three compared devices and it illustrated the Cormack-Lehane system for the laryngeal view classification. Following the lecture all participants could familiarise themselves with the equipment and practice for 30 minutes. Three skill stations were set up containing an intubation manikin (United Kingdom 3B Scientific, Weston-super-Mare, United Kingdom). The reduced movement of the cervical spine was achieved by application of a Patriot® cervical collar (Össur hf., Reykjavik, Iceland). A single-digit number was allocated to each of the three studied devices, that is, 1 for the classic Macintosh laryngoscope, 2 for the Truview Evo2 optical laryngoscope, and 3 for the Totaltrack VLM device. Each student was asked to randomly give a number between 1 and 3 and was then given the corresponding airway device to use. The maximum number of intubation attempts was limited to three per device. The novice participants of the study performed all intubations with each of the three devices. A failed intubation was defined as an attempt during which the trachea could not be intubated, for example, oesophageal tube placement, or an attempt that lasted longer than 120 seconds. Only those who failed to intubate were allowed another attempt. During direct laryngoscopy a student/participant was asked to report/describe the laryngeal inlet view using the Cormack-Lehane classification. The time required to obtain a view of the entry to the larynx (*T*_1_) and the intubation to successful ventilation time (*T*_2_) were recorded. Damage to the incisors, efficacy of intubation, and Cormack-Lehane laryngoscopic view were also assessed [14]. Gum elastic bougie was not used as an adjunct during intubation. All the obtained data was analysed using Microsoft Office Excel 2007 spreadsheet (Microsoft Corporation, Redmond, WA, USA). The Truview Evo2 laryngoscope and the Totaltrack VLM device were compared to the classic Macintosh laryngoscope which was used as a reference. Student's *t*-test was used for data analysis. Based on the previous study in order to detect a 13 s standard deviation difference (*α* = 0.05, 2-sided, *β* = 0.1), 36 participants were required [15]. The final adjusted sample size, allowing a drop-out rate of 10%, was 40 and this was the number of participants enrolled into the study. A *P* value of less than 0.05 (*P* < 0.05) was considered as statistically significant. The approval from the research ethics committee was not required for this study.

## 3. Results

The mean time required to obtain the entry-to-the-larynx view was the shortest for the Macintosh laryngoscope, 13.4 s (±2.14). This result was comparable to the one of the Truview Evo2 laryngoscope which was 14.2 s (±2.36). Furthermore this device (Truview Evo2) had the shortest intubation to successful ventilation time 35.7 s (±9.27). It was similar to the performance with the Macintosh laryngoscope (39.1 s ± 4.57) but significantly shorter than the time required to intubate and ventilate successfully with the Totaltrack VLM device (52.8 s ± 11.06) (Table 1).

There were three failed intubation attempts in the Macintosh group (the tracheal tube was inserted into the oesophagus). There were no failed intubations when the other two studied devices were used. The efficacy of intubation at first attempt was the highest for the Totaltrack VLM device (87.5%) followed by Truview Evo2 (82.5%) and the Macintosh laryngoscope (80%) (Table 2). However these results were not statistically significant (Macintosh versus Truview Evo2, *P* = 0.18, Macintosh versus Totaltrack VLM, *P* = 0.14, and Truview Evo2 versus Totaltrack VLM, *P* = 0.42).

The best view of the entry to the larynx was obtained by the Totaltrack VLM device. In this case the grade 1 laryngoscopic view was achieved in 60% of intubation attempts. When the Macintosh laryngoscope was used, grade 1 view was only achieved in 10% of attempts (Table 3): Macintosh versus Truview Evo2, *P* < 0.0001, Macintosh versus Totaltrack VLM, *P* < 0.0001, and Truview Evo2 versus Totaltrack VLM, *P* = 0.0071.

The use of the Macintosh laryngoscope was associated with the highest incidence of incisors damage (10 in 40 intubations, 25%). This was approximately twice as much as that for the Truview Evo2 laryngoscope and 5 times more than that for the Totaltrack VLM device. The latter was associated with the lowest incidence of incisor damage (Table 4). There was a moderate negative correlation between the incisor damage and intubation to successful ventilation time (*r* = −0.63405).

## 4. Discussion

Intubation with simultaneous cervical spine immobilisation remains the standard of care during management of trauma patients despite an extremely limited evidence base for this practice [16–18]. However, application of MILS during airway management may significantly impede intubation. There are devices and techniques available which may improve the laryngoscopic view and hence the ease of intubation when MILS is applied. These devices are an alternative to, the still widely used, Macintosh laryngoscope. In our study the Truview Evo2 laryngoscope and the Totaltrack VLM device were compared to the classic Macintosh laryngoscope. Both the Truview Evo2 and the Macintosh laryngoscope have been extensively studied and there is a vast amount of literature available on this. The Totaltrack VLM is a relatively new device which enables continuous ventilation of a patient before and during intubation. Its design incorporates features of both the intubating laryngeal mask airway (ILMA) and a videolaryngoscope. This may particularly be of benefit during airway management of trauma patients. In the current literature there is a lack of studies which compare intubation devices with simultaneous MILS application in the hands of novices, for example, students. Such an approach, that is, evaluation of devices in a population not previously exposed to any intubation devices, would clearly eliminate the potential source of selection bias and hence would provide more methodically robust results.

The main aim of our study was to compare three intubation devices in respect of the time required to obtain a view of the entry to the larynx and intubation to successful ventilation time. The mean *T*_1_ was prolonged for the Truview Evo2 laryngoscope as well as for the Totaltrack VLM device. The mean *T*_2_ was comparable for the Macintosh and the Truview Evo2 laryngoscopes (39.1 s and 35.7 s, resp.) but significantly prolonged for the Totaltrack VLM device (52.8 s). These results are comparable with those achieved in similar studies in a group of patients anaesthetised for elective procedures [11–13]. In a study by Maharaj et al. the Airtraq® optical laryngoscope was shown to reduce intubation time when compared to the Macintosh laryngoscope [9]. In contrary to that, Turkstra et al. did not observe the difference in intubation time (the Macintosh laryngoscope versus Airtraq). However in this study the Airtraq device was found to reduce cervical spine motion by 66% [8]. Patient's safety during airway management, which includes intubation, remains a priority. In the current practice the main aim is to adequately oxygenate a patient [19]. Despite longer times, both *T*_1_ and *T*_2_, required for the Totaltrack VLM device in the authors' opinion this device is safe as it allows continuous ventilation and hence oxygenation during intubation attempts [20].

In our study the efficacy of intubation, incidence of incisor damage, and the laryngoscopic view were also assessed. The use of alternative devices, that is, the Truview Evo2 laryngoscope and the Totaltrack VLM device, was associated with an increased efficacy of intubation (less attempts required for successful tracheal tube placement) when compared to the Macintosh laryngoscope. In our study both of the alternative devices achieved 100% efficacy. These results are similar to those described by Takahashi et al. who compared Airway Scope (AWS) to the Macintosh laryngoscope [5, 6]. Furthermore the use of the Macintosh laryngoscope was associated with the highest incidence of incisor damage. The Totaltrack VLM was the safest device in this respect. The use of alternative devices, that is, Truview Evo2 and Totaltrack VLM, achieved a better laryngoscopic view when compared to the Macintosh laryngoscope. This is especially significant for the Totaltrack VLM as this device achieved Cormack-Lehane grade 1 laryngoscopic view in 60% of intubations. In studies by Aoi et al. an alternative to the Macintosh laryngoscope was AWS and Glidescope, respectively [6, 10]. In both of the abovementioned studies, the authors achieved better laryngoscopic views when the two alternative devices, that is, AWS and Glidescope, were used. These results are analogous to those of our study which compared the Totaltrack VLM device, the Truview Evo2, and the Macintosh laryngoscopes.

The main weakness of our study design was a relatively small sample size composed of 40 participants. It is also worth noting that it is often difficult to extrapolate the results of a manikin study into the human population. This is partly because of the manikin characteristics as well as inability to replicate a significant diversity of human airway anatomy [21]. Furthermore manikin studies often provide results that are not supported by subsequent human studies or results with a variable performance of new videolaryngoscopes when compared with the Macintosh laryngoscope [22, 23].

Given the lack of high quality evidence in the current literature, it is also difficult to assess the predictive validity of manikin studies for intubation in situations where cervical spine immobilisation is required or neck movement is reduced, for example, trauma patients, although some previous studies were promising [24]. Further clinical studies are required to evaluate the effectiveness of new intubating devices in humans. Despite the abovementioned findings there is a well-recognised place for manikin studies in contemporary research [25]. The authors agree on a proposed three-stage evaluation process for a new airway device in which stage 1 would be a manikin study [26, 27].

A particular strength of our study design was that, by recruiting participants who were novices in airway skills, we reduced a confounding factor of operator's experience.

## 5. Summary

The new devices, that is, Truview Evo2 laryngoscope and the Totaltrack VLM, may be an alternative to the classic Macintosh laryngoscope during intubation of patients with suspected or present cervical spine injury when the neck mobility is reduced. This study has found that their use enables better visualisation of the entry to the larynx, a minimised risk of incisor damage during intubation, and improved rate of successful intubation.

## Figures and Tables

**Table 1:** Comparison of studied devices.

Intubating device	Time required to obtain the laryngeal inlet view (*T*_1_) (s)				Intubation to successful ventilation time (*T*_2_) (s)			
	Min	Max	Mean (SD)	*P* value	Min	Max	Mean (SD)	*P* value
Macintosh laryngoscope	3.5	30.2	13.4 (2.14)	0.12^a^	12.8	90.0	39.1 (4.57)	0.04^a^
Truview Evo2	4.0	52.5	14.2 (2.36)	<0.0001^b^	17.0	78.3	35.7 (9.27)	<0.0001^b^
Totaltrack Video Laryngeal Mask (VLM)	4.6	68.0	21.5 (6.2)	<0.0001^c^	13.8	107.2	52.8 (11.06)	<0.0001^c^

Compared intubating devices: ^a^Macintosh and Truview Evo2, ^b^Truview Evo2 and Totaltrack VLM, and ^c^Macintosh and Totaltrack VLM; SD: standard deviation; s: seconds.

**Table 2:** Efficacy of intubation.

Number of attempts	Device					
	Macintosh laryngoscope		Truview Evo2		Totaltrack VLM	
	*n*	%	*n*	%	*n*	%
1	32	80	33	82.5	35	87.5
2	33	82.5	40	100	40	100
3	37	92.5	—		—	

**Table 3:** Visualisation of the entry to the larynx.

Cormack-Lehane grade	Macintosh laryngoscope		Truview Evo2		Totaltrack VLM	
	*n*	%	*n*	%	*n*	%
1	4	10	12	30	24	60
2	26	65	24	60	9	22.5
3	10	25	4	10	7	17.5
4	0	0	0	0	0	0

**Table 4:** Incisor damage during intubation.

Device	Percentage of incisor damage
Macintosh laryngoscope	25%
Truview Evo2	12.5%
Totaltrack VLM	5%
